# Crohn’s and Colitis Canada’s 2021 Impact of COVID-19 and Inflammatory Bowel Disease in Canada: Children and Expectant Mothers With Inflammatory Bowel Disease

**DOI:** 10.1093/jcag/gwab030

**Published:** 2021-11-05

**Authors:** Eric I Benchimol, Matthew W Carroll, Rose Geist, Anne M Griffiths, James Guoxian Huang, David R Mack, Charles N Bernstein, Alain Bitton, Jennifer L Jones, Gilaad G Kaplan, M Ellen Kuenzig, Kate Lee, Mariam S Mukhtar, Sanjay K Murthy, Parul Tandon, Laura E Targownik, Joseph W Windsor, Cynthia H Seow

**Affiliations:** 1 SickKids Inflammatory Bowel Disease Centre, Division of Gastroenterology, Hepatology and Nutrition, The Hospital for Sick Children, Toronto, Ontario, Canada; 2 Child Health Evaluative Sciences, SickKids Research Institute, Toronto, Ontario, Canada; 3 ICES, Toronto, Ontario, Canada; 4 Department of Paediatrics and Institute of Health Policy, Management and Evaluation, University of Toronto, Toronto, Ontario, Canada; 5 Department of Pediatrics, University of Alberta, Edmonton, Alberta, Canada; 6 Department of Psychiatry, University of Toronto, Toronto, Ontario, Canada; 7 CHEO Inflammatory Bowel Disease Centre and Department of Pediatrics, University of Ottawa, Ottawa, Ontario, Canada; 8 Department of Internal Medicine, Max Rady College of Medicine, Rady Faculty of Health Sciences, University of Manitoba, Winnipeg, Manitoba, Canada; 9 University of Manitoba IBD Clinical and Research Centre, Winnipeg, Manitoba, Canada; 10 Department of Medicine, McGill University Health Centre, McGill University, Montreal, Quebec, Canada; 11 Department of Medicine, Dalhousie University, Halifax, Nova Scotia, Canada; 12 Department of Medicine, University of Calgary, Calgary, Alberta, Canada; 13 Department of Community Health Sciences, University of Calgary, Calgary, Alberta, Canada; 14 Crohn’s and Colitis Canada, Toronto, Ontario, Canada; 15 Department of Internal Medicine, King Abdulaziz University Hospital, Jeddah, Saudi Arabia; 16 The Ottawa Hospital IBD Centre, Department of Medicine, University of Ottawa, Ottawa, Ontario, Canada; 17 Division of Gastroenterology and Hepatology, Mount Sinai Hospital, University of Toronto, Toronto, Ontario, Canada

**Keywords:** Coronavirus disease 2019 (COVID-19), Crohn’s disease, Epidemiology, Inflammatory bowel disease, Mental health, Pediatrics, Pregnancy, SARS-CoV-2, ulcerative colitis

## Abstract

Coronavirus disease 2019 (COVID-19) in children with inflammatory bowel disease (IBD) typically results in a mild infection, similar to those without IBD. Children and adolescents have less severe manifestations of COVID-19 compared to older people, whether or not they have IBD. However, some IBD medications (in particular, corticosteroids) are associated with more severe COVID-19. During the first year of the global pandemic, more IBD care was provided with online technology, necessitated by efforts to reduce hospital and clinic visits. Additionally, non-endoscopic monitoring of inflammation has been required due to the cancellation of non-urgent procedures, resulting in longer endoscopy wait-times. In contrast, pregnant people (with and without IBD) who contract COVID-19 are at increased risk of severe manifestations, death and preterm delivery, making them a priority for severe acute respiratory syndrome coronavirus 2 protective measures and vaccination. Few studies have examined effect of COVID-19 on IBD-related disease activity in pregnant people with IBD. The pandemic has significantly affected the mental health and sense of well-being of children and their families, as well as pregnant people with IBD. These groups were much more likely to experience anxiety and depression compared with prior to the pandemic, even while concern has mostly abated regarding the effect of IBD medications and COVID-19 severity. Unfortunately, the availability of mental health care providers who specialize in people with IBD has not kept pace with the increasing demand.

**Key Messages**

- Similar to most children and adolescents, children with IBD have a milder COVID-19 disease course compared to older adults and fewer children with IBD require hospitalization for COVID-19 according to the SECURE-IBD registry; however, those on systemic corticosteroids or 5-aminosalicylates are at risk of more severe COVID-19.
- Pregnant people are at greater risk of a more severe COVID-19 disease course or birth complications. Data on the impact of COVID-19 in pregnant people with IBD are lacking.
- Since the onset of the pandemic, worsening mental health, including anxiety and depression, has been demonstrated in children, adolescents and pregnant people with IBD.
- Necessitated by protective measures and health system closures, the way in which children, adolescents and pregnant people with IBD are treated within the health system has changed. More care is provided virtually, with online telehealth clinic visits and non-endoscopy monitoring of IBD disease activity and inflammation.
- The Crohn’s and Colitis Canada COVID-19 and IBD Taskforce provided detailed guidance for children and adolescents with IBD, including instruction on protective measures, back-to-school instructions and vaccination recommendations. Guidance for pregnant people with IBD followed that of the general population.

## OVERVIEW OF COVID-19 IN CHILDREN

As of April 9, 2021, there have been over 1 million cases of coronavirus disease 2019 (COVID-19) in Canada, of which 17.7% occurred in children and adolescents under 20 years of age. Children are less severely ill with COVID-19 (1), but there are some unique epidemiologic and clinical characteristics of the disease in children.

Most children and adolescents with COVID-19 experience only mild symptoms such as cough, rhinorrhea, fatigue or intermittent fevers, with approximately 35% being asymptomatic (2). This age group comprised only 1.7% of the total 54,000 individuals hospitalized with COVID-19, and only 1.3% of the total nearly 10,000 ICU admissions. That said, some younger people have followed a more serious clinical course. To date, there have only been seven deaths in children and adolescents from COVID-19 in Canada. In the United States, children diagnosed with a chronic condition were threefold more likely to be hospitalized or admitted to an ICU compared to those without a chronic condition (3). Compared to teenagers, younger children were more likely to experience a severe COVID-19 disease course (defined as care provided in an ICU or step-down unit, or death), as were males (3).

A rare and severe vasculitic syndrome associated with COVID-19 called multisystem inflammatory syndrome in children (MIS-C) was recently described (4,5) . It can occur up to 6 weeks after infection with the severe acute respiratory syndrome coronavirus 2 (SARS-CoV-2), and more frequently affects younger children, and those of Black and Hispanic ethnicity (4). Diagnostic criteria include fever >24 h; laboratory evidence of inflammation; and evidence of severe illness requiring hospitalization with multisystem organ involvement (cardiac, renal, respiratory, hematologic, gastrointestinal, dermatologic or neurological), combined with a confirmed positive recent SARS-CoV-2 infection by PCR, serology or antigen testing, or known exposure to a suspected or confirmed case within 4 weeks of symptom onset (6). A recent study reported the incidence of MIS-C to be 2.1 per 100,000 persons under 21 years of age in the United States, with a wide geographic variation in incidence (7). Although the pathogenesis is unknown, there is similarity of this syndrome to other multisystem inflammatory vasculitis syndromes in children also triggered by other viral infections (8).

While the clinical course of COVID-19 is typically mild in children, there are some concerns disproportionately facing children and adolescents with the disease. School closures due to public health protective measures may affect socialization, learning and development. In addition, there is evidence that children are disproportionately affected by mental health concerns as a result of social isolation, pandemic-related anxiety and lack of physical activity (9). Furthermore, children may also be affected by long COVID syndrome, defined as symptoms persisting weeks to months after the start of symptoms that were suggestive of COVID-19 (10), despite an initially mild disease course (11).

## OUTCOMES OF COVID-19 IN CHILDREN WITH INFLAMMATORY BOWEL DISEASE

The first reports of COVID-19 in children with inflammatory bowel disease (IBD) were described in a publication from the Pediatric IBD Porto Group of the European Society for Pediatric Gastroenterology, Hepatology and Nutrition (12). In this case series of seven children who were confirmed or highly suspected of infection with SARS-CoV-2 before March 26, 2020, all had mild COVID-19 and did not require hospital admission despite their use of immunosuppressive medications. A larger study combined pediatric cases reported before October 1, 2020 to the Surveillance Epidemiology of Coronavirus (COVID-19) Under Research Exclusion (SECURE-IBD) registry with cases reported to the Paediatric IBD Porto Group registry (13). These two registries included a total of 209 children with IBD from 23 countries, of whom 14 were hospitalized and 2 required mechanical ventilation. As with the larger SECURE-IBD publications of adults with IBD, predictors of hospitalization in children included use of steroids and 5-aminosalicylic acids (5-ASA) medications. While steroids are known to globally inhibit the immune system, 5-ASA medications are not immunosuppressive. It is more likely that use of 5-ASA medications is a marker for poor access to more effective medications and specialist care, which may also be associated with worse COVID-19 outcomes. Children who were using anti-tumour necrosis factor monotherapy were less likely to require hospitalization compared to those who were not using anti-tumour necrosis factor monotherapy (7% versus 51%, *P* < 0.01). Children with a comorbid condition, moderate/severe active IBD and gastrointestinal symptoms were also more likely to be hospitalized. The association with 5-ASA medications remained after adjusting for disease activity (odds ratio [OR]: 4.2; 95% confidence interval [CI]: 1.3, 14.1). However, the small number of hospitalizations did not allow for more detailed multivariable regression analyses. A case series of 13 children in Texas who tested positive for SARS-CoV-2 was similarly reassuring. Six (46.2%) participants were asymptomatic. Of the remaining seven (53.8%) who developed symptoms, none required hospitalization (14). Finally, a report of COVID-19 cases in people with IBD from Italy compared rates in the first and second waves of the pandemic. The authors found an increased rate of infection in the second wave in adults, but not in children. In that study, no children were reported affected with COVID-19 in either wave (15).

At the time of writing (April 11, 2021), there were 34 children <10 years old and 614 adolescents 10 to 19 years old reported to the SECURE-IBD registry. Only 29 (4.5%) persons <20 years old were hospitalized, 3 (0.5%) required mechanical ventilation and none died. The existing literature is reassuring that children with IBD are similarly affected with COVID-19 as their non-IBD peers and are likely to experience a mild disease course. Additional research is required to confirm these initial findings, identify who might be at risk for severe COVID-19, better understand the associations between steroids and 5-ASAs with severe COVID-19, clarify why biologics are protective, determine the risk of MIS-C and better define long COVID in the pediatric IBD population.

## HEALTH SYSTEM IMPACT OF COVID-19 IN CHILDREN WITH IBD

While available information on COVID-19 outcomes in children with IBD is sparse, there are multiple studies that have evaluated the impact of the COVID-19 pandemic on the health care provided to children with IBD. Early in the pandemic, Italian centres described decreases in hospital admissions (including for newly diagnosed IBD), endoscopic re-evaluations and transfer to adult care. Few Italians with IBD stopped immunomodulators or postponed biologic infusions due to fear or uncertainty of COVID-19 outcomes (16). Another multicentre study from the United Kingdom demonstrated similarly reduced access to endoscopy and routine investigations in April 2020. In fact, over half of those newly diagnosed with IBD in that month were presumed diagnoses due to lack of access to endoscopy (17). A cross-sectional study from Israel surveyed families of children with IBD and found the majority did not perceive any major changes to their health care in the first wave of the pandemic (18). For the people with IBD in Canada, non-urgent endoscopic services were severely affected by the first wave of the pandemic, with follow-up procedures to assess therapeutic response cancelled until May to June 2020. However, since then, these semi-elective procedures have resumed and have not been subject to cancellation, even during the second and third waves when elective surgeries for adults were cancelled for specific hospitals in certain regions, often dictated by the burden of COVID-19 on ICUs.

One significant change to health services observed was the shift to virtual/online clinics. Both Italy and the United Kingdom described more than 90% of outpatient visits during the pandemic as virtual (16,17). Similar changes were made in Canada to accommodate outpatient IBD management in pediatric IBD practices. In most centres, more than 80% of outpatient visits to pediatric health centres took place virtually in 2020; but in 2021, this has begun to return towards a pre-pandemic level (p.c. centre leaders in the Canadian Children IBD Network). Families of children with chronic diseases have expressed high rates of satisfaction with virtual clinic visits (19), particularly for children who are clinically stable. Those who live in remote and rural regions have previously been used to this model of health care delivery (e.g., telehealth); however, these platforms often required the individual to travel to a local clinic or health centre to be seen. Newer models allow people with IBD to be seen from home. The families of children with Type 1 diabetes have indicated they would prefer virtual care be integrated in outpatient visits even when pandemic restrictions are removed (19). However, the clinical practice of IBD is decidedly different with physical examination (e.g., overall appearance, growth parameters, abdominal exam, perianal examination), which is still an important feature of decision-making beyond laboratory analysis.

There is concern that a shift to a virtual model of care may place some children with IBD at risk for negative outcomes. One drawback may be the inability to assess body anthropometrics in children with IBD at risk for poor weight gain or linear growth delay. A retrospective chart review from a single British centre demonstrated that children with IBD had a significantly decreased body mass index (BMI) in the time period after the national lockdown (July to November 2020). This decrease was present in children with lower BMI prior to the lockdown and was not present in normally nourished or obese children (20). Another important consideration regarding virtual versus in-person care is that individual patient–physician interactions in the absence of parents usually commences in a clinic setting during the pediatric to adult transition process; the process, including issues surrounding patient confidentiality, may be impaired by virtual visits. Patient adherence to follow-up laboratory investigations may deteriorate when they are not performed on the day of an in-person clinic visit. Finally, some specialized tests (e.g., fecal calprotectin) may require the family to pay out-of-pocket at outside laboratories while many pediatric health centres cover the cost of this test for patients. However, these drawbacks are offset by patient satisfaction, reduced travel costs and avoidance of work and school absences for medical appointments. While a hybrid model can offer benefit for the patient and family, clearly defined parameters for clinic versus virtual visits that carefully consider both personalized disease-related factors and psychosocial factors will be needed.

## MENTAL HEALTH IMPACT OF COVID-19 IN CHILDREN AND YOUTH WITH IBD

There is a growing awareness that the measures used to control the pandemic may have had an impact on mental health, especially for youth (21,22). Concern is even greater for youth with chronic medical conditions, as well as their parents and families, who already face ongoing stress and challenges associated with coping with the medical condition and its treatment. Since early in the pandemic, the unintended consequence of social avoidance has been social disruption. Adolescent development depends on the creation of growing social comfort beyond the family. These connections are central to the ongoing development of a strong personal identity and sense of self on the road to autonomy, which is key to promoting mental health (23). Moreover, there is a growing literature on the relationship between social development and brain maturation in adolescence (24). It is generally accepted that, for young people, optimal learning occurs in a school environment, and that school is a major source of social growth. In this setting, students experience friendship, natural competition and stimulation from one another; all of this promotes the process of learning and enriches outcomes. However, COVID-19 safety precautions have required young people to stay at home and avoid person-to-person socialization. Online and at-home learning is being encouraged as an important measure of safety. Without the social contact that comes with an in-school learning environment, what can we expect when it comes to the impact on learning and other outcomes, especially when this social disruption occurs during adolescence?

In a cross-sectional survey of German youth with IBD (mainly adolescents) and their parents, Reinsch et al. found that parents experienced increased fear of their children contracting COVID-19 in general, but especially in the school environment (25). In fact, school was the environment most feared with respect to contracting the SARS-CoV-2 virus. At the same time, and in the same study, the youth with IBD generally coped well with their IBD and adhered to their IBD medications as well as to the recommended hygiene protocols (25). These findings suggest that the increased levels of fear were more related to the pandemic than to ongoing health concerns. Further, in a cross-sectional telephone survey in Israel, Dorfman *et al.* found that youth with IBD (mainly adolescents) were very worried about COVID-19 because of their belief that they had an increased susceptibility to contracting the virus (18). This fear resulted in these people voluntarily increasing their avoidance of attending school beyond the ministry requirements of their country. Unfortunately, despite the need, mental health support may be more difficult to access during the pandemic. Major mental illness frequently first occurs in adolescence, which is a particularly vulnerable time. Additionally, pediatric-onset IBD is associated with the development of psychiatric disorder along the lifespan (26,27). Therefore, it is especially important for young people and their families to be aware that pediatric IBD clinicians can be important sources of mental health support. These team members are not only sources of the most reliable medical information, but they are also trusted authority figures. Pediatric IBD teams should encourage both individuals with IBD and their parents to discuss their beliefs and fears associated with the pandemic. Communication of accurate information regarding IBD and COVID-19 will help to reduce pandemic-related fears; further, promoting an atmosphere in which individuals with IBD and their families can discuss their fears will also help to facilitate the identification of a possible psychiatric disorder, where referral to a mental health provider is critical.

For all youth, and especially for youth with IBD, socialization should be encouraged as much as possible, while being mindful of the necessary safety guidelines. Individuals with IBD and their parents may not realize they can turn to their IBD care providers for mental health support, and so opportunities should be provided as needed. Provision of both the correct information about COVID-19, as well as an overall atmosphere in which fears may be openly discussed, will go a long way to supporting the mental health of youth with IBD, especially during these trying times.

## COVID-19 AND PREGNANT PEOPLE WITH IBD

The association of COVID-19 infection and negative outcomes of pregnancy is well described. Early in the pandemic, an association between SARS-CoV-2 infection and preterm births by caesarean section was described (28). In addition, studies from the United States (29,30) and Sweden (31) have demonstrated pregnant people with COVID-19 were more than fourfold more likely to be admitted to the ICU and require mechanical ventilation. A recent systematic review determined that COVID-19 in pregnancy was associated with an increased risk of preeclampsia (OR: 1.33; 95% CI: 1.03, 1.73), preterm birth (OR: 1.83; 95% CI: 1.38, 2.39) and stillbirth (OR: 2.11; 95% CI: 1.14, 3.90) (32). Having severe COVID-19 (compared to mild COVID-19) was associated with preeclampsia (OR: 4.16; 95% CI: 1.55, 11.15), gestational diabetes (OR: 1.99; 95% CI: 1.09, 3.64), preterm birth (OR: 4.29; 95% CI: 2.41, 7.63) and low birth weight (OR: 1.89; 95% CI: 1.14, 3.12) (32). Another systematic review examined the care provided to pregnant people and their infants internationally during the COVID-19 pandemic compared to pre-pandemic; it found that the pandemic was associated with a significantly increased risk of maternal death (OR: 1.37; 95% CI: 1.22, 1.53) and stillbirth (OR: 1.28; 95% CI: 1.07, 1.54) (33). However, in the pandemic era, there was no increased risk of preterm birth (OR: 0.81; 95% CI: 0.67, 0.97), preterm birth before 28 weeks gestation (OR: 0.84: 95% CI: 0.45, 1.53), postpartum hemorrhage (OR: 1.02; 95% CI 0.87, 1.19), NICU admission for the infant (OR: 0.90; 95% CI: 0.80, 1.01) or low birthweight (OR: 0.99; 95% CI: 0.90, 1.08). However, the pandemic era was associated with increased postnatal depression, as measured by an increased Edinburgh Postnatal Depression Scale score (pooled mean difference: 0.42; 95% CI: 0.02, 0.81) (33).

Very few studies have assessed the impact of COVID-19 on pregnant people with IBD. A registry of IBD prenatal care provided in 13 British hospitals found no confirmed cases of COVID-19 among 244 pregnant people with IBD, and a very low rate of negative pregnancy outcomes (34). In that study, the majority of health care encounters occurred by telephone (68.2%, compared to 3% prior to the pandemic). There was no increase in the number of IBD-related questions to their advice-line, compared to before the pandemic (34). Preliminary results from a Canadian survey of pregnant people with IBD also found no confirmed cases of COVID-19, although 8 of 29 participants had symptoms suspicious for COVID-19 (35). Respondents with Crohn’s disease (not ulcerative colitis) experienced increased symptoms of anxiety, depression and stress compared to before the pandemic (35). Recruitment for this study is ongoing with more detailed results anticipated (p.c. Tandon).

## VACCINES IN SPECIAL POPULATIONS

As vaccines roll out in adult populations, plans for vaccinating children and adolescents in Canada are still waiting for clinical trial data. All individuals included in the clinical trials leading to vaccine approval were over 16 years of age. Pfizer recently reported 100% vaccine efficacy in a clinical trial of 2260 adolescents aged 12 to 15 years (36), leading to Health Canada’s approval of the Pfizer vaccine for this age group. Side effects were generally mild and included injection-site pain, headaches, fever and fatigue. Teenagers were found to have comparable levels of virus-neutralizing antibodies 1 month after the second dose as study participants aged 16 to 25 in the adult trial. Results are expected soon from a similar trial of the Moderna mRNA vaccine in adolescents aged 12 to 17 years. AstraZeneca recently began a study of its vaccine among those aged 6 to 17 years in Britain, and Pfizer has begun a trial in infants as young as 6 months old.

Similar to the pediatric population, pregnant people were also excluded from initial vaccine trials; however, a recent analysis of 35,691 pregnant people from multiple large registries of mRNA vaccinated individuals reported rates of adverse pregnancy and neonatal outcomes that are similar to vaccination studies conducted before the COVID-19 pandemic, including a 13.9% rate of pregnancy loss, a 9.4% rate of preterm birth and a 3.2% rate of small size for gestational age, with no neonatal deaths (37). A recent cohort study further demonstrated that vaccine-induced antibody titres in pregnant and lactating people following COVID-19 vaccination are comparable to those in non-pregnant individuals and are significantly higher than titres induced by SARS-CoV-2 infection itself (38). Studies have also found that anti-SARS-CoV-2 IgG antibodies following COVID-19 mRNA vaccination are present in umbilical cord blood and breast milk, with efficient transplacental antibody transfer, thus conferring passive immunization (38–40). Vaccination earlier in pregnancy further resulted in higher antibody concentrations in the infants (39,40). While early data demonstrated hesitancy among pregnant people to receive the COVID-vaccine (41), more recent data show better acceptance in this group, particularly among people with better education and employment (42). This highlights the need for ongoing vaccine education and strategies to manage vaccine hesitancy.

## CROHN’S AND COLITIS CANADA COVID-19 AND IBD TASKFORCE RECOMMENDATIONS

In general, our recommendations for children with IBD are similar to the recommendations for adults with IBD. In addition to strict adherence to regional public health measures, caution should be exhibited by children with IBD to avoid direct indoor contact with non-family members, adhere to physical distancing and masking guidelines and have awareness of patient risk profile recommendations described earlier. We recognize the importance of in-person school attendance to the developmental and psychosocial well-being of children and adolescents. We therefore recommend in-person school attendance for children with IBD unless indicated otherwise by regional public health authorities. However, children and adolescents with severe active inflammation, those using systemic corticosteroids and/or with moderate–severe malnutrition should not attend school in-person due to the association of these factors with severe COVID-19 disease. School attendance may be resumed when the youth’s IBD is in remission, steroid doses are tapered below 0.5 mg/kg/day (and 20 mg/day) and malnutrition has been treated (43).

The COVID-19 IBD Taskforce did not make specific recommendations for pregnant people with IBD. They should follow the guidance provided to all individuals with IBD, as well as regional public health guidelines.
